# Association between Egg Consumption and Dementia in Chinese Adults

**DOI:** 10.3390/nu16193340

**Published:** 2024-10-01

**Authors:** Precious O. Igbinigie, Ruoling Chen, Jie Tang, Alexandru Dregan, Jiaqian Yin, Dev Acharya, Rizwan Nadim, Anthony Chen, Zhongliang Bai, Farzad Amirabdollahian

**Affiliations:** 1 Faculty of Education, Health and Wellbeing, University of Wolverhampton, Wolverhampton WV1 1LY, UKr.chen@wlv.ac.uk (R.C.);; 2 School of Public Health, Guangzhou Medical University, Guangzhou 511436, China; gytanjie@163.com; 3 Division of Academic Psychiatry, Institute of Psychiatry, Psychology and Neuroscience, King’s College London, London SE5 8AF, UK; 4 Lifespan and Population Health, School of Medicine, University of Nottingham, Nottingham NG7 2UH, UK; 5 School of Health Services Management, Anhui Medical University, Hefei 230032, China

**Keywords:** egg consumption, China, case-control study, dementia, dietary intakes, older adults

## Abstract

Background/Objectives: The association between egg consumption and dementia is unclear. We carried out a population-based case-control study in China to determine the independent association of egg consumption with dementia. Methods: We randomly recruited 233 participants with dementia and 233 without dementia from the community health service clinics and the dementia management system in Guangzhou, China to examine their dietary intakes over the past two years and other risk factors for chronic diseases. Egg consumption was categorised by frequency as Non-consuming/<monthly, Monthly, Weekly, Daily or ≥Twice a day. Results: Participants with dementia, compared to controls, were more likely to consume eggs at Monthly (15.5% vs. 8.6%) but less likely to consume at Daily (28.3% vs. 41.6%). The age-adjusted odds ratio (OR) of dementia was 1.76 (95% CI 1.10–2.84) in participants who consumed eggs Weekly and 4.34 (2.16–8.72) in Monthly consumption compared to Daily. However, no significant associations were found for those Non-consuming/<monthly 1.35 (0.71–2.56) and ≥Twice a day 3.49 (0.83–14.67). After further adjustments, including gender, education, family income, smoking, alcohol consumption, dietary intakes (including red meats, poultry, fish, vegetables and fruits), cardiovascular disease and other co-morbidities, the corresponding ORs were 2.10 (1.10–4.02), 4.82 (1.90–12.27), 0.73 (0.29–1.88) and 4.16 (0.80–21.63), respectively. Among participants who consumed eggs Monthly, Weekly or Daily, an inverse association between egg consumption and dementia was observed; the multiple adjusted OR of dementia was 0.48 (0.30–0.76) per average increment in egg consumption. Compared to Monthly consumption, the OR for Weekly consumption was 0.44 (0.18–1.08) and 0.22 (0.08–0.59) for Daily consumption. Conclusions: This study suggests that Daily egg consumption could help reduce the risk of dementia, while uncertainties regarding the association of non-consuming/<monthly or ≥Twice a day consumption with dementia warrant further research.

## 1. Introduction 

Dementia has become an important public health issue due to the increase in the ageing population in the world. More than 55 million people are affected by dementia globally, with an annual incident rate of 10 million, and the number of people with dementia is expected to increase to 152 million by 2050 [1]. Currently, there is no known cure for dementia, making primary prevention a priority in public health to reduce the incidence of dementia and delay its progression. There has been increasing growth of research examining the association between dietary intake and dementia risk and prognosis, such as fish consumption [2,3,4], while other modifiable risk factors [5], e.g., smoking, depression [6] and air pollution [7], are targeted for dementia prevention. Previous studies have shown that the Mediterranean diet (MeDi), which includes eggs, may reduce the risk of dementia in the population [2]. Eggs are nutrient-dense food and a good source of choline, folate, vitamin D, iodine, B vitamins and high-quality protein [8,9]. They are a natural functional food because they contain highly bioavailable carotenoids, such as lutein and zeaxanthin, and other nutrients, which include nutraceuticals known to protect against chronic disease. Many people incorporate chicken eggs into their daily diets because they are an affordable source of protein [10,11] and other nutrients [8,9]. However, it is important to note that despite their nutritional benefits, egg consumption has been associated with elevated levels of blood cholesterol [12], which can be detrimental to health. There has been increased research on the health effects of egg consumption in the population. Six cohort studies in the United States, which consisted of 29,615 adults with a median follow-up of 17.5 years, showed that each additional half-egg consumed per day was significantly associated with a higher risk of incident cardiovascular disease (CVD) (adjusted hazard ratio [HR] 1.06, 95% CI 1.03–1.10) and all-cause mortality (adjusted HR, 1.08, 95% CI 1.04–1.11) [13]. To examine the impact of egg consumption on the incidence of type 2 diabetes (T2D), Drouin-Chartier et al. [14] analysed data from three large US cohort studies and performed a systematic review of prospective cohort studies worldwide. They found that each 1 egg/d was significantly associated with a higher risk of T2D in Americans but not among European or Asian populations. There have been a few studies undertaken to assess the association between egg consumption and cognitive impairment (CI), with inconsistent findings; some showed that adequate egg consumption would reduce the risk of developing CI [15,16,17,18], while others found no association [19,20]. Research on the association between egg consumption and dementia risk is scarce, and only a couple of studies were undertaken in Western countries, leaving uncertainty regarding the impact of egg consumption on dementia [21]. Furthermore, the findings from studies conducted in high-income countries (HICs) may not be generable to low- and middle-income countries (LMICs), where dietary intakes, nutritional statuses and patterns of disease and risk factors differ from those in HICs. 

China is the largest LMIC and has the largest number of people with dementia in the world. It is the number one producer of eggs, producing about 40% of the world’s total eggs [22]. China is a major consumer of eggs [23], and its per capita egg consumption is twice the world’s average [24]. Around 65% of Chinese people report eating more than four eggs weekly [22], which is higher than in other countries, such as the UK, Netherlands, Chile and Brazil [25]. However, little is known about the association between egg consumption and dementia risk in China. No studies have been conducted to assess the impact of the daily consumption of eggs on the risk of dementia in Chinese people. In this paper, we examined data from a population-based case-control study to determine the association between egg consumption and dementia.

## 2. Methods

We conducted a case-control study in Guangzhou, China, to investigate new risk factors for dementia [7]. From July to October 2020, we randomly recruited participants for the case-control study through community health service clinics (CHSCs) in Guangzhou City and the dementia management system (DMS), which is managed by Huiai Hospital (also known as Guangzhou Brain Hospital), to follow up patients with dementia in Guangzhou. At Guangzhou CHSC, doctors would use the Mini-Mental State Examination (MMSE) (Chinese version and validated) to identify people with cognitive dysfunction (MCI) [26] and then refer them to Huiai Hospital or other advanced hospitals for further examination. At the hospital, doctors employ a standard process to diagnose dementia [27], including single-domain cognitive assessment (memory, language, visual and execution assessments), behavioral assessment via the Neuropsychiatric Questionnaire (NPI), functional assessment including instrumental life function (IADL) and basic life function (BADL) and brain imaging such as Magnetic Resonance Imaging (MRI). The CHSCs transfer information about dementia patients who have been clinically diagnosed to the DMS and the physical examination service system (PES) in Guangzhou, which is linked to each of the CHSCs. The DMS is utilised to manage patients with dementia in Guangzhou. Annual follow-ups are conducted for all patients in the system who have already been diagnosed with dementia using the 10th revision of the International Statistical Classification of Diseases and Related Health Problems (ICD) diagnostic criteria (ICD-10). Through the DMS, we subsequently enrolled cases from the Community Neurology Management Department at Huiai Hospital. Medical examinations were provided by the Physical Examination Department at HuiH. The inclusion criteria for participants in the case group were individuals clinically diagnosed with dementia. We selected the controls through CHSCs during an annual health check for older residents. Their data were then transferred to the PES and managed by the Health Committee of Guangzhou for storage and management. We randomly recruited the controls through PES, ensuring the exclusion of potential dementia cases based on cognitive function tests as screening. The inclusion criteria for participants in the control group were individuals not clinically diagnosed with dementia. In both the case and control samples, we included those aged over 50 years. Their diagnoses of dementia and non-dementia were further confirmed by a neurologist from our Guangzhou research team using ICD-10 criteria, excluding individuals who had been diagnosed with epilepsy, meningioma or any other tumours, and any severe psychiatric illness (e.g., schizophrenia and paranoid psychosis) from this study. We successfully recruited 233 cases and 233 controls for an interview in this study. All dementia patients in our study had been diagnosed within the past year and were ambulatory during interviews.

Before commencing the interview, we sought and obtained informed consent from each participant. In participants where an individual was unable to provide informed consent due to disability or limited educational level, their next of kin or caregivers would provide assent for the interview. Our interview team conducted face-to-face interviews with each participant in separate and safe locations within HuiH or other designated places including participant’s home. Participants were encouraged to have a family member or caregiver present during the interview for support. The primary interview material utilised was a validated Chinese version of the General Health and Risk Factor Questionnaire [3,4,6,7]. The General Health and Risk Factors Questionnaire collects data on socio-demographics, lifestyles, social networks, environmental exposures, histories of diseases and medication, dietary intake and others. During the interview, all participants were asked about the frequency of egg consumption over the past two years and categorized into five groups: (i) Non-consuming or less than once a month, (ii) ≥Monthly and <Once a week (i.e., monthly), (iii) ≥Weekly and <Once a day (i.e., weekly), (iv) ≥Daily and <Twice a day (i.e., daily) and (v) ≥Twice a day. Each interview lasted approximately 45 min. After completing the questionnaire, its integrity was reviewed, and the data were encoded.

### Data Analysis

We described the characteristics of participants using mean ± SD for continuous variables and percentage (%) for categorical variables and examined their differences between two groups of dementia and non-dementia using a *t*-test for continuous variables and a Chi-square test for categorical variables. We also examined the relationship of egg consumption with other dietary intakes apart from the basic characteristics. A binary logistic regression model was employed to assess the association between egg consumption and dementia. In the logistic regression model, we calculated odds ratios (ORs) and their 95% confidence intervals (CIs) for dementia in participants with different levels of egg consumption, adjusting for important confounders including age, gender, socioeconomic status, lifestyles, co-morbidities and other dietary intakes. We initially chose Daily egg consumption as the reference group for analysis since it has been associated with the most benefits for cardiovascular diseases in Chinese adults [28]. Then, in a sensitivity analysis, we additionally used each of the different levels of egg consumption as the reference group to test the association between egg consumption and dementia. All statistical analyses were performed using IBM SPSS Statistics version 28 (IBM Co., Armonk, NY, USA).

## 3. Results

Of the 466 participants, the mean age was 73.6 years (SD 9.5), ranging from 52 to 100 years (interquartile [IQR] of 67–87 years), 63.5% were women, and 57.7% had an educational level of ⩽junior high school. Table 1 shows detailed characteristics of participants with and without dementia. Compared to those without dementia, participants with dementia were more likely to be older, have lower levels of education and family income, be widowed or divorced/never married, smoke, drink no/less alcohol, have coronary heart disease or stroke, have a higher cardiovascular disease score (derived from hypertension, high blood cholesterol, diabetes, coronary heart disease and stroke) and have high levels of head injuries, chronic bronchitis, depression, migraine and Parkinson’s disease. About their dietary intake over the past two years, they were more likely to consume red meats (pork, beef and lamb) but less likely to eat poultry meat, fish, vegetables and fruits (Table 1). Other variables listed in Table 1 showed no significant differences between the two groups. Among the 466 participants, 2.8% consumed eggs at ≥Twice a day, 35.0% Daily, 36.7% Weekly, 12.0% Monthly and 13.5% Non-consuming/<monthly. We also examined differences in the basic characteristics of patients across the five levels of egg consumption. Significant age differences were observed, with participants consuming eggs at ≥Twice a day being the oldest and those consuming eggs Monthly being the youngest. Participants with higher socioeconomic status (education and family income) had increased egg consumption. Participants consuming eggs Weekly had the highest level of alcohol consumption. Depression was most prevalent among participants consuming eggs at ≥Twice a day. Furthermore, participants with chronic kidney disease (CKD) tended to consume fewer eggs (trend *p* = 0.003). There were borderline significant differences in smoking habits across the five levels of egg consumption, with participants consuming eggs Weekly or ≥Twice a day being more likely to smoke regularly than others.

Table 2 displays the dietary intakes of pork, beef, lamb, poultry (chicken/duck), fish, vegetables and fruits, and their combined subgroups across five levels of egg consumption. Chi-square tests showed significant differences in each of these dietary intakes and combined subgroups across the five levels of egg consumption. Moreover, the linear-by-linear association test revealed that these dietary intakes and combined subgroups had a significantly positive association with egg consumption, except for lamb consumption (Table 2).

Other factors listed in Table 1 showed no significant differences across the five levels of egg consumption.

Table 3 presents the number, % and odds ratio of dementia at different levels of egg consumption among participants with and without dementia. Among people with dementia, the percentage of egg consumption at Daily was smaller, but the percentages of egg consumption Weekly or Monthly were higher than those of participants without dementia. The age-adjusted OR of dementia significantly increased with egg consumption Weekly (1.76, 95% CI 1.10–2.84) and Monthly (4.34, 2.16–8.72) compared to Daily consumption, while the ORs for egg consumption at Non-consuming/<monthly and at ≥Twice a day were not statistically significant (Table 3).

Table 4 shows the multiple-adjusted odds ratios of dementia at different levels of egg consumption. After adjusting for age, gender, education and family income (Model 1), the ORs of dementia for Weekly, Monthly, Non-consuming/<monthly and ≥Twice a day egg consumption were reduced compared to those in the age-adjusted analysis (Table 3), but the ORs for Weekly and Monthly consumption remained significantly increased. After further adjusting for marital status, smoking, alcohol consumption, and co-morbidities (Model 2), the ORs for Weekly and Monthly egg consumption increased compared to those in Model 1, but the ORs for Non-consuming/<monthly and ≥Twice a day remained not significant (Table 4). Additional adjustments for other dietary intakes (Model 3) did not substantially change these ORs compared to Model 2. Specifically, there were significantly increased ORs for Weekly and Monthly egg consumption but non-significant ORs for Non-consuming/<monthly and ≥Twice a day egg consumption.

Based on the data from Table 3 and Table 4, and after excluding participants with egg consumption at ≥Twice a day and Non-consuming/<monthly for the analysis, we found a trend of increasing OR for dementia in relation to egg consumption from Daily to Weekly and Monthly. The average increment in OR across the three levels of egg consumption was 1.88 (1.33–2.65), 2.11 (1.46–3.04) and 2.08 (1.31–3.29) in Models 1–3, respectively.

Table 5 displays the multiple-adjusted ORs for dementia using different levels of egg consumption as references for analysis. When “≥Twice a day” was taken as the reference group, none of the other levels of egg consumption showed a significant OR, probably due to its small number of participants. Upon combining the groups of “≥Twice a day” with “Daily” as the reference for analysis, the Model 3-adjusted ORs were 1.87 (1.00–3.50) for Weekly, 4.31 (1.73–10.75) for Monthly and 0.67 (0.26–1.71) for Non-consuming/<monthly. When “Weekly” was taken as the reference, egg consumption Daily was significantly and inversely associated with dementia, as well as Non-consuming/<monthly, but the increased OR for Monthly egg consumption was not significant. Similar findings were observed when “Monthly” was used as the reference. When “Non-consuming/<monthly” was taken as the reference, the increased ORs for egg consumption Weekly and Monthly were significant, but not Daily and “≥Twice a day. However, upon analysis with a combination of “Non-consuming/<monthly” and “Monthly” as a reference, none of the other levels of egg consumption revealed a significant OR.

In analysing egg consumption at Monthly as the reference (Table 5), we further examined the trend of increased egg consumption associated with the risk of dementia after excluding those two groups categorised as “≥Twice a day” and “Non-consuming/<monthly”. The data from Monthly, Weekly and Daily egg consumption demonstrated a significantly reduced odds of dementia per average increment in egg consumption (Model 3-adjusted OR 0.48, 0.30–0.76). The categorial data analysis suggested that, compared to Monthly egg consumption, the Model 3-adjusted OR for Weekly egg consumption was 0.44 (0.18–1.08) (*p* = 0.073) and for Daily egg consumption was 0.22 (0.08–0.59) (*p* = 0.002), with an overall trend *p* < 0.01.

## 4. Discussion 

Our population-based case-control study examined the association between egg consumption and dementia. This study demonstrated that the odds of dementia increased with decreased consumption of eggs from Daily to Weekly to Monthly, while the odds of dementia in those consuming eggs ≥ Twice a day or those who were Non-consuming/<monthly were not significantly increased or reduced. Such an inverse association between egg consumption and dementia was independent of important confounding factors, including age, educational level, smoking, dietary intake of red meats, poultry, fish, vegetables and fruits and co-morbidities.

Despite the current literature lacking research on egg consumption associated with dementia, several studies have investigated the association with cognitive decline or impairment (CI) in older people [15,16,17,18,19,20]. An et al. [20] analysed data from 2816 older adults aged 60 years or older from the National Health and Nutrition Examination Survey (NHANES) 2011–2012 and 2013–2014 waves. Cognitive assessments included the Consortium to Establish a Registry for Alzheimer’s Disease Word List Learning Test (CERAD-WL), Word List Recall Test (CERAD-DR), Animal Fluency Test (AF) and Digit Symbol Substitution Test (DSST). Their findings indicated that neither the consumption status of whole eggs nor the quantity consumed daily was associated with cognitive test scores in older adults. However, other studies [16,17,29] have shown that the consumption of eggs could be beneficial for cognitive functioning. Sukik et al. [17] examined data from 4852 participants aged 55 years or older from the China Health and Nutrition Survey (CHNS). The CHES study collected data on dietary egg intake for each participant through 24-h dietary recalls over 3 consecutive days during home visits between 1991 and 2006 and assessed the cognitive function of the participants in 1997, 2000, 2004 and 2006. The authors found that egg intake was positively associated with global cognitive function, and in fully adjusted models, the regression coefficients across the quartiles of egg intake were 0, 0.11 (95% CI −0.28–0.51), 0.79 (95% CI 0.36–1.22) and 0.92 (95% CI 0.43–1.41), respectively. Compared to non-consumers, those with higher egg consumption (4th quartile) had an adjusted OR of 0.93 (0.74–1.19) for self-reported poor memory and 0.84 (0.69–1.02) for self-reported memory decline, both being not statistically significant. Kritz-Silverstein and Bettencourt [16] analysed data from a cohort of 617 men and 898 women aged 60 and older from the Rancho Bernardo Cohort, who were followed for 16.3 years. They found that egg intake at baseline was significantly associated with better cognitive function measured at follow-up in men (performance on Buschke total (*p* = 0.04), long-term (*p* = 0.02) and short-term (*p* = 0.05) recall but not in women (*p*-values > 0.05). Li et al. [29] examined data from a cohort of 9028 participants aged ≥ 60 years from the Zhejiang Ageing and Health Cohort Study without cognitive impairment at baseline and followed up for 6 years with three waves of measurements using the Mini-Mental State Examination (MMSE) for CI. The authors used log-binomial regression models for repeated measures with the Generalized Estimating Equations (GEE) method to assess the longitudinal effect of egg consumption on the risk of cognitive impairment. The authors found that participants who increased egg consumption had a reduced relative risk (RR) of CI compared to non-consumers or those who consumed eggs less than Weekly; the multiple adjusted RR of CI was 0.82 (0.76–0.89) for participants consuming 0.1–2.9 eggs/week, 0.91 (0.84–0.99) for those consuming 3.0–5.9 eggs/week, and 0.95 (0.86–1.04) for those consuming ≥ 6.0 eggs/week.

Few studies have been conducted to investigate the association between egg consumption and dementia. In Italy, Nicoli et al. [30] examined data from 1390 participants aged ≥ 80 years from a cross-sectional study in the Varese province and found that the adjusted OR of dementia in the middle tertile of egg consumption compared to the lowest tertile was 0.60 (0.47–0.77), while the OR in the highest tertile of egg consumption was 0.63 (0.38–1.04). The authors further analysed data from 512 participants in the cohort follow-up and found that the corresponding HRs were 0.68 (0.39–1.17) and 0.85 (0.64–1.14), suggesting no significant association. In Finland, Ylilauri et al. [21] carried out a cohort study of 2497 men aged 42–60 y, followed up for 21.9 years, and observed that each additional 0.5 eggs (27 g)/d was associated with a hazard ratio (HR) of 0.89 (95% CI 0.78–1.01) but not significantly. In Spain, Margara-Escudero et al. [31] anlaysed data from 25,015 participants aged 30–70 years from the European Prospective Investigation into Cancer and Nutrition (EPIC)-Spain Dementia Cohort. Cohort participants were recruited between 1992 and 1996 for the baseline survey and followed up for a mean of 21.5 years. The authors found no association between egg consumption and dementia; the adjusted HR for the 4th quartile vs. the 1st quartile of egg consumption was 1.05 (0.85–1.31). After dividing the population by adherence to the relative Mediterranean diet (rMED) score, they observed an inverse association between egg consumption and dementia in participants with low adherence to the rMED score (HR in Q4 vs. Q1: 0.52, 95% CI 0.30–0.90). But there was no association between participants having medium adherence (1.20, 0.89–1.62) and high adherence to the rMED score (0.93, 0.61–1.39). Our study in China revealed a significant and inverse association between egg consumption and dementia among participants who consumed eggs Monthly, Weekly and Daily. In other words, our study demonstrated that the risk of dementia was significantly reduced in these adults who consumed eggs Daily. This protective effect of Daily consumption was consistently observed regardless of whether Monthly or Weekly consumption was taken for comparison (Table 5).

Our study found that participants who consumed eggs at a frequency of ≥Twice a day might have an increased OR of dementia compared to those who consumed eggs at a frequency of Non-consuming/<monthly (Table 5) or participants who consumed eggs Daily (Table 4), approaching statistical significance. The finding suggests a possible link between excessive egg consumption and dementia risk, likely due to the high cholesterol content of eggs, with each egg containing ~200 mg of cholesterol. Previous studies using randomized controlled trials (RCTs) data [12] have revealed that individuals who consumed more than four whole eggs per week experienced greater elevations in blood total cholesterol, HDL cholesterol and LDL cholesterol compared to those who consumed equivalent amounts of egg substitutes. Animal studies involving mice, rats and rabbits have demonstrated an association between high cholesterol intake and AD-type pathologies [32,33,34]. Some human studies have also shown an association between dietary cholesterol intake and lower cognitive performance [35,36]. Furthermore, recent population-based cohort research found a positive association between blood cholesterol levels and incident dementia [37]. Our study observed an increased OR of dementia with the consumption of eggs ≥Twice a day, but it did not reach conventional statistical significance, which was probably due to the small number of participants in this category of egg consumption. Nonetheless, the potential impact of excessive egg consumption on dementia warrants further investigation. 

Our study did not find a significant increase in the OR of dementia among participants who reported non-consuming/<monthly over the past two years. There is no research examining this low level of egg consumption in relation to dementia [30]. However, previous research examining cognitive impairment in China found a relation to non-consumers or <weekly consumers; the risk of CI was significantly increased in comparison to those consuming 0.1–2.9 eggs/week or 3.0–5.9 eggs/week [29]. Also, such a group of never/rarely consuming eggs was found to have an increased risk of CVD, particularly among older people [28]. In China, eggs are a popular food due to their nutritional value, affordability and ease of preparation [22]. Despite this, our study found that 13.5% of older adults did not eat eggs or consumed them less than monthly during the two-year period. The reasons for this low consumption could be attributed to poverty. Our data showed that participants with lower family income had reduced egg consumption. These individuals who did not eat eggs over the past two years also had decreased consumption of other nutrient-rich foods such as meats, fish, vegetables and fruits (Table 2). It is worth noting that they may represent a specific subset of the older population, warranting further investigation.

Our study demonstrated that participants who consumed eggs Daily had a reduced odds ratio of dementia compared to those who consumed eggs Monthly or Weekly, regardless of the confounders adjusted for in the analysis. The protective effect of Daily egg consumption against dementia in older adults may be attributed to the presence of bioactive compounds and numerous other nutrients such as high-quality protein, unsaturated fatty acids and vitamins [38]. The nutrient-dense nature of eggs makes them a valuable source of energy, particularly for individuals at risk of malnutrition, such as older adults [39]. Previous research from RCT data [12] indicated that compared to individuals consuming ≤ 4 whole eggs per week, those consuming > 4 whole eggs per week did not experience elevated blood pressure, lipids and lipoproteins. This suggests that the increased egg consumption in our study would not be likely to contribute to cardiovascular risk, which thereby increases the incidence of dementia. Bioactive compounds like lutein, zeaxanthin and choline found in eggs may have beneficial effects on intestinal cholesterol absorption. On the other hand, due to the high levels of protein in eggs, increased egg consumption may delay sarcopenia in older adults [40], while sarcopenia could be associated with an increased risk of dementia [41].

Available evidence indicates that brain inflammation is frequently present in the pathology of dementia. This inflammation, caused by misfolded proteins, such as tau proteins, amyloid beta and alpha-synuclein, could activate microglia toll-like receptors (TLRs) and trigger an innate immune response, which in turn produces inflammatory mediators [42]. Furthermore, neuroinflammation harms the blood–brain barrier’s integrity, increasing the risk of dementia. Consumption of foods such as eggs, which contain antioxidants, choline and omega-3 fatty acids, has been linked to a decrease in systemic inflammation [43,44]. The bioactive components in eggs have anti-inflammatory qualities and may, therefore, contribute to the prevention of dementia [9,45]. Consumption of eggs has also been found to lower the risk of cardiovascular disease in Asian populations [28,46]. Qin et al. [28] examined data from a cohort study of half a million participants in China and revealed that daily egg consumption, compared to non-consuming, was associated with a lower risk of CVD (HR 0.89, 0.87–0.92) [28]. Egg’s components, including antioxidants and anti-inflammatory qualities, as well as their potential to lower the CVD risk, could help reduce the risk of dementia. Previous studies [3,47,48] have also found that the consumption of fish, vegetables and fruits can reduce the risk of dementia since these dietary intakes contain antioxidants and omega-3 fatty acids. The data of our study showed that there were positive associations between these dietary intakes and egg consumption (Table 2). The finding that daily egg consumption reduces dementia risk in our study is in line with the results of previous studies examining the impact of fish, vegetables and fruits consumption on dementia [3,47,48].

Our study examining data from this population-based case-control study in China identified that eating one egg per day could reduce the risk of dementia. The study findings contribute to the development of better policies and improve public health aimed at reducing the burden of disease by promoting egg consumption among older adults.

### Strengths and Limitations of This Study

To the best of our knowledge, this study is the first to examine the association between egg consumption and dementia in China, a country with the largest number of people with dementia and the highest egg consumption worldwide. It is also the first to report a significant association between daily egg consumption and a reduced risk of all types of dementia in the world. Our study produces new insights suggesting that daily egg consumption may be associated with a reduced risk of dementia. We included important confounders for adjustment, such as educational level, smoking, alcohol consumption, CVD, depression and consumption of red meats, fish, vegetables and fruits apart from age, thereby minimising residual effects. Additionally, we performed sensitivity analyses using different levels of egg consumption as references, ensuring the robustness of our findings. This study has limitations. First, there were significant age differences between the case and control groups due to the lack of individual age matching in the case-control design for data collection. However, in the data analysis, we adjusted for age as a continuous variable, thereby minimising the residual effect of age on the association between egg consumption and dementia risk. Furthermore, we performed separate data analysis by age of <75 years (mean age was 62.3 in people with dementia vs. 65.1 in people without dementia) and ≥75 years (81.8 vs. 75.3) and found that the results were similar to those from the overall data analysis; for example, the data from Monthly, Weekly to Daily egg consumption showed a significantly reduced odds of dementia per average increment in egg consumption from Monthly, Weekly to Daily in the age group of <75 years (Model 3-adjusted OR 0.22, 0.08–0.61) and in the age group of ≥75 years (0.45, 0.22–0.93). Second, our food frequency questionnaire collected data on the frequency of egg consumption but did not measure the quantity of eggs consumed at each level of the frequency. It may have diluted the association we identified. Previous research suggests that the daily consumption of eggs among Chinese adults usually amounts to 0.76 egg/day [28]. We analysed the frequencies of egg consumption to determine the association between egg intake and dementia and found that Daily egg consumption was associated with a lower risk of dementia in Chinese adults. Third, the case-control design of our study limits the ability to refer causality inference in the association between egg consumption and dementia. However, the observed association between increased egg consumption from Monthly to Daily and reduced risk of dementia is consistent with findings from a recently published study in Western populations [49]. Pan et al. examined data from 1024 older Americans with a mean age of 81.4 who were followed up for 6.7 years in the Rush Memory and Aging Project cohort and found that the risk of Alzheimer’s dementia was significantly reduced with Weekly consumption of >1 egg/wk (adjusted hazard ratio 0.53, 95% CI 0.34–0.83) and ≥2 eggs/wk (0.53, 0.35–0.81), compared to those who consumed ≤1 egg/wk [49]. The trend for the protective effect of egg consumption on dementia is consistent with the findings of our study. Nevertheless, cohort studies in China are required to further examine the protective effect of Daily egg consumption on dementia.

## 5. Conclusions

In conclusion, our study suggests that among Chinese adults who had regular consumption of eggs on a Monthly, Weekly or Daily basis, increased consumption of eggs is significantly associated with a reduced risk of dementia. Specially, Daily consumption of eggs appears to be protective against dementia. However, the impacts of consuming eggs at a frequency of ≥Twice a day or Non-consuming/<monthly on dementia risk remain unclear, warranting further research.

## Figures and Tables

**Table 1:** Characteristics of participants in Guangzhou case-control study, China.

			Dementia				
Variable	All Participants		Participants with Dementia		Participants without Dementia		*p*
	n = 466, (%)		n = 233, (%)		n = 233, (%)		
Sociodemographic and lifestyle							
Age (years)							
Mean (SD)	73.6	9.5	77.2	10.5	70.0	6.6	<0.001
Gender							
Women	296	63.5	153	65.7	143	61.4	0.336
Men	170	36.5	80	34.3	90	38.6	
Educational level							
University and above	50	10.7	20	8.6	30	12.9	0.001
Senior high school	60	13.0	21	9.0	39	16.7	
Junior high school	118	25.4	55	23.6	63	27.0	
Primary school	151	32.3	79	33.9	72	30.9	
Family income per year (RMB)							
≥100,000	20	4.3	16	6.9	4	1.7	0.001
70,000–<100,000	50	10.7	26	11.2	24	10.3	
50,000–<70,000	72	15.5	29	12.4	43	18.5	
30,000–<50,000	163	35.0	69	29.6	94	40.3	
10,000–<30,000	144	30.9	80	34.3	64	27.5	
<10,000	17	3.6	13	5.6	4	1.7	
Marriage							
Married or cohabiting	300	64.4	122	52.4	178	76.4	<0.001
Widow	132	28.3	89	38.2	43	18.5	
Divorced/Never married	32	6.9	22	9.4	10	4.3	
Missing ^†^	2	0.4	0	0.0	2	0.9	
Smoking							
No	380	81.5	183	78.5	197	84.5	0.002
Occasionally/formally	48	10.3	35	15.0	13	5.6	
Regularly	38	8.2	15	6.4	23	9.9	
Drinking any types of alcohol over the past 2 years							
Non-consuming or less than once per month	440	94.4	225	96.6	215	92.3	0.044
More than once a month	26	5.6	8	3.4	18	7.7	
Cardiovascular disease and risk factors							
Hypertension status							
No	219	47.0	102	43.8	117	50.2	0.164
Yes	247	53.0	131	56.2	116	49.8	
High blood cholesterol							
No	400	85.8	198	85.0	202	86.7	0.301
Yes	60	12.9	34	14.6	26	11.2	
Missing ^†^	6	1.3	1	0.4	5	2.1	
Diabetes							
No	374	80.3	184	79.0	190	81.5	0.485
Yes	92	19.7	49	21.0	43	18.5	
Coronary heart disease							
No	422	90.6	204	87.6	218	93.6	0.036
Yes	43	9.2	28	12.0	15	6.4	
Missing ^†^	1	0.2	1	0.4	0	0.0	
Stroke							
No	404	86.7	175	75.1	229	98.3	<0.001
Yes	59	12.7	55	23.6	4	1.7	
Missing ^†^	3	0.6	3	1.3	0	0	
Total score from the above five CVD variables							
Nos > median (score 1.0) and %	136	29.2	90	38.6	46	19.7	<0.001 ^&^
Kidney disease							
No	448	96.1	223	95.7	225	96.1	0.631
Yes	18	3.9	10	4.3	8	3.9	
Head injury							
No	437	93.8	211	90.6	226	97.0	0.004
Yes	29	6.2	22	9.4	7	3.0	
Chronic bronchitis							
No	412	88.4	187	80.3	225	96.6	<0.001
Yes	39	8.4	32	13.7	7	3.0	
Missing ^†^	15	3.2	14	6.0	1	0.4	
Depression							
No	431	92.5	201	86.3	230	98.7	<0.001
Yes	20	4.3	19	8.2	1	0.4	
Missing	15	3.2	13	5.6	2	0.9	
Migraine							
No	417	89.5	201	86.3	216	92.7	0.032
Yes	48	10.3	31	13.3	17	7.3	
Missing ^†^	1	0.2	1	0.4	0	0.0	
Parkinson’s disease							
No	410	88.0	179	76.8	231	99.1	<0.001
Yes	27	5.8	26	11.2	1	0.4	
Missing ^†^	29	6.2	28	12.0	1	0.4	
Dietary intake in the past two years							
Consumption of Pork							
≥Twice a day	45	9.7	35	15.0	10	4.3	<0.001
Daily	148	31.8	72	30.9	76	32.6	
Weekly	200	42.9	85	36.5	115	49.4	
Monthly	41	8.8	28	12.0	13	5.6	
Non-consuming or <Monthly	32	6.9	13	5.6	19	8.2	
Consumption of Beef							
≥Twice a day	2	0.4	2	0.9	0	0.0	<0.001 ^‡^
Daily	12	2.6	10	4.3	2	0.9	
Weekly	76	16.3	54	23.2	22	9.4	
Monthly	95	20.4	37	15.9	58	24.9	
Non-consuming or <Monthly	281	60.3	130	55.8	151	64.8	
Consumption of Lamb							
≥Twice a day	4	0.9	4	1.7	0	0.0	0.054 ^‡^
Daily	11	2.4	8	3.4	3	1.3	
Weekly	33	7.1	17	7.3	16	6.9	
Monthly	57	12.2	22	9.4	35	15.0	
Non-consuming or <Monthly	361	77.5	182	78.1	179	76.8	
Total score from above score							
Nos > median (6.0) and %	175	37.6	106	45.5	69	29.9	<0.001 ^&^
Consumption of Poultry Meat							
≥Twice a day	19	4.1	13	5.6	6	2.6	0.003
Daily	70	15.0	27	11.6	43	18.5	
Weekly	234	50.2	107	45.9	127	54.5	
Monthly	81	17.4	53	22.7	28	12.0	
Non-consuming or <Monthly	62	13.3	33	14.2	29	12.4	
Consumption of Fish							
≥Twice a day	37	7.9	21	9.0	16	6.9	<0.001
Daily	91	19.5	37	15.9	54	23.2	
Weekly	242	51.9	101	43.3	141	60.5	
Monthly	61	13.1	44	18.9	17	7.3	
Non-consuming or <Monthly	35	7.5	30	12.9	5	2.1	
Total score from above two Poultry and Fish							
Nos > median (6.0) and %	122	26.2	54	23.2	68	29.2	0.140
Consumption of Vegetables							
≥Twice a day	190	40.8	99	42.5	91	39.1	<0.001
Daily	193	41.1	74	31.8	119	51.1	
Weekly	55	11.8	37	15.9	18	7.7	
Monthly	17	3.6	15	6.4	2	0.9	
Non-consuming or <Monthly	11	2.4	8	3.4	3	1.3	
Consumption of Fruits							
≥Twice a day	37	7.9	24	10.3	13	5.6	<0.001
Daily	186	39.9	83	35.6	103	44.2	
Weekly	173	37.1	73	31.3	100	42.9	
Monthly	46	9.9	32	13.7	14	6.0	
Non-consuming or <Monthly	24	5.2	21	9.0	3	1.3	
Total score from above two vegetable and fruit							
Nos > median (8.0) and %	116	24.9	57	24.5	94	25.3	0.046

^†^ *p*-Values in the Chi-square test are calculated based on the available data, not including missing data, if any. ^‡^ With Fisher–Freeman–Halton Exact Test. ^&^ Non-parametric Kuskal–Walllis H test.

**Table 2:** Dietary intake among participants in Guangzhou case-control study, China.

Variable	Consumption of Eggs in the Past Two Years										
Dietary Intake in the Past Two Years ^†^	Non-Consuming or <Monthly		Monthly		Weekly		Daily		≥Twice a Day		*p* ^‡^
(Frequency)	n	%	n	%	n	%	n	%	n	%	
Consumption of Pork											
Non-consuming or <Monthly	10	15.9	4	7.1	8	4.7	8	4.9	2	15.4	0.001
Monthly	5	7.9	17	30.4	8	4.7	11	6.7	0	0.0	
Weekly	16	25.4	16	28.6	115	67.3	53	32.5	0	0.0	
Daily	23	36.5	11	19.6	34	23.0	79	48.5	1	7.7	
≥Twice a day	9	14.3	8	14.3	6	3.5	12	7.4	10	76.9	
Consumption of Beef											
Non-consuming or <Monthly	51	81.0	40	71.4	92	53.8	91	55.8	7	53.8	0.002
Monthly	4	6.3	12	21.4	34	19.9	44	27.0	1	7.7	
Weekly	7	11.1	3	5.4	40	23.4	21	12.9	5	38.5	
Daily	1	1.6	1	1.8	4	2.3	6	3.7	0	0.0	
≥Twice a day	0	0.0	0	0.0	1	0.6	1	0.6	0	0.0	
Consumption of Lamb											
Non-consuming or <Monthly	52	82.5	48	85.7	127	74.3	122	74.8	12	92.3	0.563
Monthly	3	4.8	7	12.5	22	12.9	25	15.3	0	0.0	
Weekly	3	4.8	0	0.0	21	63.6	9	27.3	0	0.0	
Daily	4	6.33	1	1.8	0	0.0	6	3.7	0	0.0	
≥Twice a day	1	1.6	0	0.0	1	0.6	1	0.6	1	0.6	
Total score from above score											
Nos > median											
Nos > median and %	18	28.6	14	25.0	68	39.8	65	39.9	10	76.9	0.004
Consumption of Poultry Meat											
Non-consuming or <Monthly	22	34.9	12	21.4	11	6.4	16	9.8	1	7.7	<0.001
Monthly	11	17.5	27	48.2	23	13.5	20	12.3	0	0.0	
Weekly	17	27.0	14	25.0	123	71.9					
Daily	8	12.7	3	5.4	10	5.8	45	27.6	4	30.8	
≥Twice a day	5	7.9	0	0.0	4	2.3	4	2.5	6	46.2	
Consumption of Fish											
Non-consuming or <Monthly	6	9.5	2	3.6	2	1.2	1	0.6	0	0.0	0.007
Monthly	6	9.5	8	14.3	2	1.2	1	0.6	0	0.0	
Weekly	2	3.2	4	7.1	39	22.8	10	6.1	0	0.00	
Daily	23	36.5	17	30.4	65	38.0	83	50.9	5	38.5	
≥Twice a day	26	41.3	25	44.6	63	36.8	68	41.7	8	61.5	
Total score from above two Poultry and Fish											
Nos > median											
Nos > median and %	16	25.4	11	19.6	22	12.9	63	38.7	10	76.9	<0.001
Consumption of Vegetables											
Non-consuming or <Monthly	6	9.5	2	3.6	2	1.2	1	0.6	0	0.0	<0.001
Monthly	6	9.5	8	14.3	2	1.2	1	0.6	0	0.0	
Weekly	2	3.2	4	7.1	39	22.8	10	6.1	0	0.00	
Daily	23	36.5	17	30.4	65	38.0	83	50.9	5	38.5	
≥Twice a day	26	41.3	25	44.6	63	36.8	68	41.7	8	61.5	
Consumption of Fruits											
Non-consuming or <Monthly	9	14.3	5	8.9	5	2.9	4	2.5	1	7.7	<0.001
Monthly	9	14.3	14	25.0	13	7.6	9	5.5	1	7.7	
Weekly	19	30.2	15	26.8	78	45.6	60	36.8	1	7.7	
Daily	17	27.0	17	30.4	68	36.6	80	49.1	4	30.8	
≥Twice a day	9	14.3	5	8.9	7	4.1	10	6.1	6	46.2	
Total score from above two vegetable and fruit											
Nos > median											
Nos > median and %	11	17.5	14	25.0	40	23.4	42	25.8	9	69.2	0.003

^†^ in the Chi-square test for each of these variables, where the Fisher–Freeman–Halton Exact Test was used, the *p* value for all variables of dietary intakes and combined sores was <0.001, except *p* = 0.003 for beef consumption and 0.002 for lamb consumption. ^‡^ *p* value for linear-by-linear association in the Chi-square test.

**Table 3:** Number, % and crude OR of cases in different levels of egg consumption: Guangzhou case-control study, China.

Egg Consumption in the Past 2 Years	With Dementia		Without Dementia			Age-Adjusted Analysis		
	N = 233	%	N = 233	%	*p* ^†^	OR ^‡^	95% CI	*p*
≥Twice a day	10	4.3	3	0.60	0.005	3.49	0.83–14.67	0.088
Daily	66	28.3	97	41.6		Ref		
Weekly	90	38.6	81	34.8		1.76	1.10–2.84	0.019
Monthly	36	15.5	20	8.6		4.34	2.16–8.72	0.000
Non-consuming or <Monthly	31	13.3	32	13.7		1.35	0.71–2.56	0.362

^†^ Fisher–Freeman–Halton Exact Test. ^‡^ OR adjusted for age.

**Table 4:** Multiple adjusted OR of dementia in different levels of egg consumption: Guangzhou case-control study, China.

Egg Consumption in the Past 2 Years	Model 1			Model 2			Model 3		
	OR^1^	95% CI	*p*	OR^2^	95% CI	*p*	OR^3^	95% CI	*p*
≥Twice a day	3.39	0.82–14.03	0.092	2.32	0.46–11.80	0.311	4.16	0.80–21.63	0.090
Daily	Ref			Ref			Ref		
Weekly	1.66	1.00–2.74	0.048	2.04	1.11–3.72	0.021	2.10	1.10–4.02	0.024
Monthly	3.86	1.86–8.02	0.000	5.11	2.13–12.24	0.000	4.82	1.90–12.27	0.001
Non-consuming or <Monthly	1.14	0.59–2.22	0.697	1.03	0.46–2.34	0.939	0.73	0.29–1.88	0.518

OR^1^ in Model 1: Adjusted for age, gender, education and family income; OR^2^ in Model 2: Adjusted for age, gender and education, family income, marital status, smoking, alcohol consumption, CVD score (hypertension, high blood cholesterol, diabetes, coronary heart disease and stroke), kidney disease, chronic bronchitis, head hurt, Parkinson’s disease and depression; OR^3^ in Model 3: Adjusted for age, gender, education, family income, marital status, smoking, alcohol consumption, CVD score (hypertension, high blood cholesterol, diabetes, coronary heart disease and stroke), kidney disease, chronic bronchitis, head hurt, Parkinson’s disease and depression, as well as the scores for red meat consumption (pork, beef and lamb), poultry and fish consumptions and vegetable and fruit consumption.

**Table 5:** Sensitivity analysis for OR of dementia in participants with different levels of egg consumption.

Egg Consumption in the Past 2 Years	“≥Twice a Day” as Ref			“Weekly” as Ref			“Monthly” as Ref			“Non-Consuming or <Monthly” as Ref		
	OR^3^	95% CI	*p*	OR^3^	95% CI	*p*	OR^3^	95% CI	*p*	OR^3^	95% CI	*p*
≥Twice a day	Ref			1.98	0.38–10.25	0.418	0.86	0.15–5.12	0.870	5.67	0.92–35.08	0.062
Daily	0.24	0.05–1.25	0.090	0.48	0.25–0.91	0.024	0.21	0.08–0.53	0.001	1.36	0.53–3.50	0.518
Weekly	0.51	0.10–2.63	0.418	Ref			0.44	0.18–1.04	0.060	2.87	1.16–7.11	0.022
Monthly	1.16	0.20–6.88	0.870	2.29	0.96–5.44	0.060	Ref			6.58	2.16–20.01	0.001
Non-consuming or <Monthly	0.18	0.03–1.09	0.062	0.35	0.14–0.86	0.022	0.15	0.05–0.46	0.001	Ref		

OR^3^ in Model 3: Adjusted for age, gender, education, family income, marital status, smoking, alcohol consumption, CVD score (hypertension, high blood cholesterol, diabetes, coronary heart disease and stroke), kidney disease, chronic bronchitis, head hurt, Parkinson’s disease, depression, and the score of red meat consumptions (pork, beef and lamb).

## Data Availability

The original contributions presented in the study are included in the article, further inquiries can be directed to the corresponding author.
